# Characteristics of clinical isolates of nontuberculous mycobacteria in Java-Indonesia: A multicenter study

**DOI:** 10.1371/journal.pntd.0011007

**Published:** 2022-12-27

**Authors:** Leli Saptawati, Widana Primaningtyas, Paramasari Dirgahayu, Yusup Subagio Sutanto, Brian Wasita, Betty Suryawati, Titik Nuryastuti, Ari Probandari

**Affiliations:** 1 Department of Microbiology, Faculty of Medicine, Universitas Sebelas Maret, Surakarta, Indonesia; 2 Doctoral Program in Medicine, Faculty of Medicine, Universitas Sebelas Maret, Surakarta, Indonesia; 3 Department of Microbiology, Moewardi Teaching Hospital, Surakarta, Indonesia; 4 Faculty of Medicine, Universitas Sebelas Maret, Surakarta, Indonesia; 5 Department of Parasitology and Micology, Faculty of Medicine, Universitas Sebelas Maret, Surakarta,Indonesia; 6 Department of Pulmonology, Moewardi Teaching Hospital, Surakarta, Indonesia; 7 Department of Anatomical Pathology, Faculty of Medicine, Universitas Sebelas Maret, Surakarta, Indonesia; 8 Department of Microbiology, Faculty of Medicine, Public Health and Nursing, Universitas Gadjah Mada, Yogyakarta, Indonesia; 9 Indonesia Biofilm Research Collaboration Center, Universitas Gadjah Mada—BRIN, Yogyakarta, Indonesia; 10 Department of Public Health, Faculty of Medicine, Universitas Sebelas Maret, Surakarta, Indonesia; Indian Institute of Science Education and Research Bhopal, INDIA

## Abstract

**Background:**

Nontuberculous mycobacterial (NTM) lung infections are a major public health concern. Diagnosis of NTM-pulmonary disease (NTM-PD) is difficult because its clinical, microbiological, and radiological features resemble to those of pulmonary tuberculosis (TB), leading to misdiagnosis. Identification at the species level is essential for diagnosis and determination of therapy, which is currently not performed routinely in Indonesian laboratories.

**Methodology and principal findings:**

From January 2020 to May 2021, 94 NTM isolates were collected from three TB referral centers in Java Province. Species were identified using matrix-assisted laser desorption-ionization time-of-flight mass spectrometry (MALDI-TOF MS). Tests were performed to determine antibiotic susceptibility, biofilm formation ability, sliding motility characteristics, and the ability to adhere to and invade pneumocytes. After identifying the species of all the isolates, we found nine groups of NTMs: *M*. *fortuitum* group 51% (48/94), *M*. *abscessus* 38.3% (36/94), *M*. *intracellulare* 3.1% (3/94), *M*. *neoaurum* 2.1% (2/94), *M*. *chelonae* 1.1% (1/94), *M*. *gordonae* 1.1% (1/94), *M*. *szulgai* 1.1% (1/94), *M*. *mucogenicum* 1.1% (1/94), and *M*. *arupense* 1.1% (1/94). Amikacin was the most effective antibiotic against *M*. *fortuitum* group and *M*. *abscessus*. The *M*. *fortuitum* group was significantly better at forming biofilms than *M*. *abscessus*, but both had the same sliding motility capability. The ability of the *M*. *fortuitum* group to adhere to and invade pneumocytes was better than that of *M*. *abscessus*, with the number isolates of the *M*. *fortuitum* group capable of superior adhesion and invasion to that of *M*. *abscessus*.

**Conclusions/Significance:**

This study shows that *M*. *fortuitum* group and *M*. *abscessus* were the most common NTM found in Java, Indonesia. The *M*. *fortuitum* group and *M*. *abscessus* were the most susceptible to amikacin; therefore, this was the empirical treatment of choice. The ability to form biofilms is directly proportional to the ability to adhere to and invade pneumocytes but not to the susceptibility profile or sliding motility characteristics.

## Author summary

Nontuberculous mycobacterial (NTM) infection is a serious public health problem that can cause various clinical manifestations of infection, including NTM-pulmonary lung disease (NTM-PD). NTM-PD infections are often misdiagnosed as pulmonary tuberculosis, leading to underestimation of the incidence of NTM infections, particularly in countries where tuberculosis is endemic. The lack of routine laboratory services in Indonesia in terms of species identification and susceptibility to NTM is a factor complicating diagnosis and patient management. These factors have led to a lack of adequate information on NTM infections in Indonesia. Research on species identification and susceptibility testing is important to help clinicians better manage NTM infections. This study was conducted on 94 clinical isolates of NTM from Java Province, and it is the first study in Indonesia to identify NTM to the species level using matrix-assisted laser desorption ionization time-of-flight mass spectrometry. The most common species found were *M*. *fortuitum* group and *M*. *abscessus*. *M*. *abscessus* was more resistant to ciprofloxacin, moxifloxacin, amikacin, trimethoprim/sulfamethoxazole, and doxycycline than *M*. *fortuitum* group. Amikacin is the most effective antibiotic and can be used as the empirical antibiotic of choice.

## Introduction

There are three groups of *Mycobacteria* that are pathogenic to humans: the *M*. *tuberculosis complex* (MTC) that causes tuberculosis, *M*. *leprae* and *M*. *lepromatosis* that cause leprosy, and nontuberculous mycobacteria (NTM) [1]. NTM are divided into rapidly growing mycobacteria (RGM), which can form colonies within 7 days, and slowly growing mycobacteria (SGM), which take more than 7 days to form new colonies [2]. To date, the extent of the global burden of NTM infections remains unclear. There are various challenges related to describing morbidity and mortality rates, incidence and prevalence, trends, and distribution of NTM infections at regional, national, and global levels [3]. In most countries, cases of NTM infections do not receive special attention and are not routinely reported, so epidemiological data are still very limited, especially in countries with limited resources [4,5]. Most reports of NTM infections originate from countries where tuberculosis (TB) is not endemic and only a few from TB endemic [6]. In TB-endemic countries, the lack of awareness of NTM infection is increasing [4]. Some countries might have data related to NTM infection through the implementation of TB programs, but this may lead to underestimation because cases of NTM infections that do not cause clinical manifestations of TB are not recognized [3]. NTM-pulmonary disease (NTM-PD) is the most common clinical manifestation of NTM infection and accounts for 80–90% of all NTM-associated diseases [7]. Structural lung disease (cystic fibrosis, chronic obstructive pulmonary disease, etc.) and immunosuppression (human immunodeficiency virus [HIV], transplantation, etc.) are the main predisposing factors for NTM-PD [7]. The prevalence of NTM-PD is steadily increasing, making it one of the most severe public health issues [8]. The availability of microbiological and molecular laboratory diagnostic methods for NTM has contributed to the increasing detection of NTM infections in clinical samples [9]. The mortality rate of NTM infections is also increasing [5]. A study in Indonesia conducted in Surakarta, Central Java, showed that 15% of 1,636 positive acid-fast bacilli cultures were NTM isolates [10]. However, to date, there are no routine services for species identification and NTM susceptibility testing.

The clinical, microbiological, and radiological features of NTM-PD are similar to those of pulmonary TB, making diagnosis difficult [7]. Therefore, this leads to the misdiagnosis of NTM-PD as pulmonary TB and an underestimation of the incidence of NTM infection in many TB-endemic countries [9]. Culture examinations of sputum samples are among the microbiological investigations required to establish a diagnosis of NTM-PD [11]. In addition, the treatment of NTM-PD differs from that of TB. Each NTM species has a specific antibiotic therapy regimen. Inadequate NTM management leads to therapeutic failure, increased drug toxicity, and the development of antibiotic resistance [12]. Antibiotic susceptibility testing should be conducted against agents that are believed to be effective against each type of NTM species [13]. Most NTM species are resistant to anti-TB drugs [9]. Species identification is critical for diagnosis and determination of antibiotic treatment [14].

Previous studies have reported a significant relationship between the ability to form biofilms and the clinical manifestations of infection. This indicates that the ability to form biofilms is an important virulence factor [15]. The formation of biofilms allows NTM to be more resistant to various antibiotics, persist longer in the environment, and colonize the respiratory tract, which has the potential to cause infections [16]. Some NTM strains are also capable of sliding motility, i.e. the ability to shift to relocate to another site, thereby accelerating the spread of biofilms to the surfaces of neighboring objects [17]. The ability to form biofilms and perform sliding motility is different in each species [18]. The presence of long-chain mycolic acids in the genus *Mycobacterium* is related to the hydrophobic nature of bacteria, which affects their ability to adhere to surfaces [15]. This is the first NTM study in Indonesia that covers a large area and identifies NTM at the species level. The aim of this exploratory, cross-sectional was to determine the species type, susceptibility level, biofilm formation, sliding motility characteristics, and adhesion and invasion ability of NTM isolates from three provinces of Java Island, Indonesia (West Java, Central Java, and East Java).

## Methods

### Ethics statement

The Medical and Health Research Ethics Committee of the Faculty of Medicine, Universitas Sebelas Maret, reviewed and approved the present study protocol (approval no. 76/UN27.06.6.1/KEP/EC/2021).

### Clinical samples and study designs

This was an exploratory cross-sectional study. NTM cultures were isolated from patient sputum using the BACTEC MGIT 960 system (Becton Dickinson), followed by a rapid test to detect MPT 64 antigen using SD Bioline TB Ag MPT 64 (Standard Diagnostics, Seoul, South Korea). The isolate was confirmed as NTM if the culture showed positive results and MPT 64 antigen detection showed negative results. A total of 94 NTM isolates were collected from sputum specimens from the West Java region: the Gunawan Pulmonary Hospital (RSPG) Bogor, the National TB referral center; The Central Java region: the Central Java Province Health Laboratory and Medical Device Testing Center in Semarang; and the TB referral center in the East Java region, the Surabaya Health Laboratory Center. Each NTM isolate was obtained from a different patient. The isolates were collected between January 2020 and May 2021. The isolates were stored in skim milk at -80°C. Prior to the experiments, subcultures were performed on Lowenstein-Jensen (LJ) medium and tested for purity.

### NTM species identification

Species identification was performed for all samples, by using matrix-assisted laser desorption-ionization time-of-flight mass spectrometry (MALDI-TOF MS) (Vitek MS *Mycobacterium*/*Nocardia* Kit, bioMérieux SA, Marcy L’Étoile, France). Briefly, 1 μL of NTM colonies grown on LJ medium was transferred to a tube containing glass beads and 70% ethanol, vortexed for 15 min, and incubated for additional 10 min at room temperature. The dried pellets were then uniformly dispersed in 70% formic acid and acetonitrile. After centrifugation, 1 μL of the supernatant was transferred to a slide. After drying, 1 μL of CHCA matrix (bioMérieux SA, Marcy L’Eoile, France) was applied and allowed to dry before the analysis. Identification was performed automatically. For calibration and quality control, *Escherichia coli* (ATCC 8739; American Type Culture Collection, Manassas, VA, USA) was used for each run according to the manufacturer’s protocol [19].

### Susceptibility assay

The test procedure was performed following the method of Li et al. (2013), with slight modifications [20]. Susceptibility assays for all 94 isolates to seven antibiotics: ciprofloxacin (Dexa, Indonesia), moxifloxacin HCl (Novell, Indonesia), clarithromycin (Sanbe, Indonesia), amikacin sulfate (Phapros, Indonesia), impenem and cilastatin (Bernofarm, Indonesia), trimethoprim/sulfamethoxazole (Mersifarma Tirmaku Mercusuana, Indonesia), and doxycycline hyclate (Yarindo, Indonesia), were performed using the broth microdilution method in 96-well microtiter plates (Corning Costar, Sigma-Aldrich, Missouri, USA). Antibiotics were prepared at a final concentration of 1000 μg/mL and the stock was stored at 4°C. A broth microdilution technique using Middlebrook 7H9 broth was used to determine the minimum inhibitory concentration (MIC). All tests were performed at least thrice for each isolate. The *Mycobacterial* inocula were prepared from NTM cultures on LJ slants.The inocula were then adjusted to McFarland standard 0.5 with saline and then adjusted to a dilution of 1:20 with Middlebrook 7H9 supplement (7H9-S) (7H9 broth +10% ADC + 0.5% glycerol). Antibiotics were serially diluted two-fold in 100 μL 7H9-S. As a positive control, wells were filled with Middlebrook 7H9 broth and an inoculum without antibiotics. As a negative control, wells were filled with Middlebrook 7H9 broth. MIC was defined as the lowest drug concentration that prevented bacterial growth, as indicated by the absence of turbidity in the tubes. On day 7, the MIC was determined. The MIC breakpoints of the drugs were interpreted according to the Clinical and Laboratory Standards Institute guidelines (M24-A2). Standard strains of *Staphylococcus aureus* ATCC 29213 and *Pseudomonas aeruginosa* ATCC 27853 were used as quality controls according to Clinical and Laboratory Standards Institute recommendations [13].

### Biofilm assay

The quantitative biofilm testing procedure was performed on all samples according to the method of Hassan et al. (2011) with slight modifications [21]; 2 μL of bacteria grown for 24 hours at 37°C in Middlebrook 7H9 medium were inoculated into the wells of 96 well-flat bottom polystyrene tissue culture-treated plates (Corning Costar, Sigma-Aldrich, Missouri, USA), filled with 198 μL Middlebrook 7H9 medium. The negative control organism (*S*. *epidermidis* ATCC 12228) was incubated, diluted, and added to a tissue culture plate [22]. The wells of the negative control contained sterile broth. The plates were incubated at 37°C for seven days. After incubation, the contents of each well were gently tapped out. The wells were washed three times with 0.2 mL phosphate buffer saline (PBS) (pH 7.2). This removed all free-floating bacteria. Biofilms formed by bacteria adhering to the wells were stained with crystal violet (0.1%). Aquadest was used three times to remove excess staining. The biofilm was resuspended in 200 μL of 5% isopropanol, and absorbance was measured using a microplate reader (Bio-Rad Benchmark, New Delhi) at a wavelength of 595 nm. The experiment was performed six times. Bacterial optical density (OD) was calculated based on the average of at least three replicates with nearly the same value. The optical density cutoff value (ODc) was calculated based on the average OD of the negative control organism. Biofilm production was evaluated according to the criteria described by Stepanovic et al. Strong biofilms are indicated when the OD value of the bacteria is > 4× the ODc value, moderate biofilms are indicated when the bacterial OD value is > 2× the ODc value and ≤ 4× the ODc value, weak biofilms are indicated when the bacterial OD value is > ODc and the bacterial OD value is ≤ 2× ODc, and negative biofilms are indicated when the bacterial OD is ≤ ODc [23].

### Scanning electron microscopy (SEM)

Scanning electron microscopy (SEM) observations were performed on the biofilm state of the *M*. *fortuitum* group and *M*. *absessus* isolated by following the method described by Nuryastuti et al. (2018) with slight modifications [24]. NTM biofilms were formed on sterile polyvinyl chloride coverslips (0.13–17 mm thick and 13 mm in diameter) in 12-well microtiter plates (Corning Costar, Sigma-Aldrich, Missouri, USA), and 198 μL Middlebrook 7H9 medium was added. The plates were incubated for 7 days at 35–37°C. The wells overgrown with biofilm were washed twice with PBS (0.1 M and pH 7.2). The coverslips were removed, rehydrated with ethanol (70% concentration for 10 min and then 96% for 10 min), and air-dried overnight in a desiccator. The coverslip was coated twice with platinum- vanadium using a sputter ion (Bal-Tec SCD 005) and then bonded to a double-sided carbon tape for examination using SEM (JEOL JED-2300, Japan).

### Sliding motility assay

The sliding motility test was performed as described by Esteban et al. (2008) [25] on 31 NTM isolates, consisting of 16 isolates from the *M*. *fortutium* group and 15 isolates of *M*. *abscessus*. A total of 3 μL NTM isolate at OD 6000.6 (2.7 × 10^5^ colony forming units [CFU]) was added to the center of a plate containing motility medium, consisting of Middlebrook 7H9 medium with 0.3% agar without supplements. The inoculated medium was incubated at 37°C in a 5% CO_2_ atmosphere for 16 days. The diameter of the bacterial growth was measured on days 4, 8, 12, and 16 using a digital caliper. At least three independent sliding motility experiments were performed, and results are presented as mean ± standard deviation (SD).

### Adherence and invasion assay

NTM adhesion and invasion into the A549 cell line (human type II pneumocytes) were performed on *M*. *fortuitum* group and *M*. *abscessus* isolates, which formed a strong biofilm following the protocol in previous publication by Eijkelkamp et al. (2011), with modifications [26]. Cell lines were grown in Dulbecco’s modified Eagle’s medium (DMEM; Invitrogen, Australia) supplemented with 10% fetal bovine serum (FBS; Bovogen, Australia), streptomycin (100 μg/mL), and 2 mM glutamine. The cell monolayer was examined microscopically before experiments to ensure >95% confluency. Washed A549 monolayers in 12-well tissue culture plates were then infected with a bacterial inoculum containing 1 × 10^7^ CFU. The inoculum number was determined using a viable cell-counting assay. After 4 h of incubation at 37°C, the culture medium was removed, and the cell monolayers were detached from the plate by treatment with 0.25% trypsin. The eucaryotic cells were then lysed by the addition of 200 μL sterile 0.025% Triton X-100 and diluted 1000×. Twenty microliters of the suspension were plated on Middlebrook 7H9 agar to determine the number of CFU of adherent bacteria per well. After incubation for 10 days, the number of bacterial colonies was determined. Data for the adherence assay were obtained from at least three independent experiments, and each assay was repeated twice. The number of bacteria capable of adhesion and invasion is expressed as mean ± standard error (SE).

### Statistical analysis

All experiments were performed in triplicate. The chi-square test was used to analyze the differences in proportions. The t-test was used to analyze differences between the groups. However, because the data were not normal, the Kruskal–Wallis test was used to compare the data from more than two groups, and the Mann–Whitney test was used to compare the data from two groups. Statistical significance was set at *p<0*.*05*.

## Results

### Distribution of NTM Species

**Fig 1:**
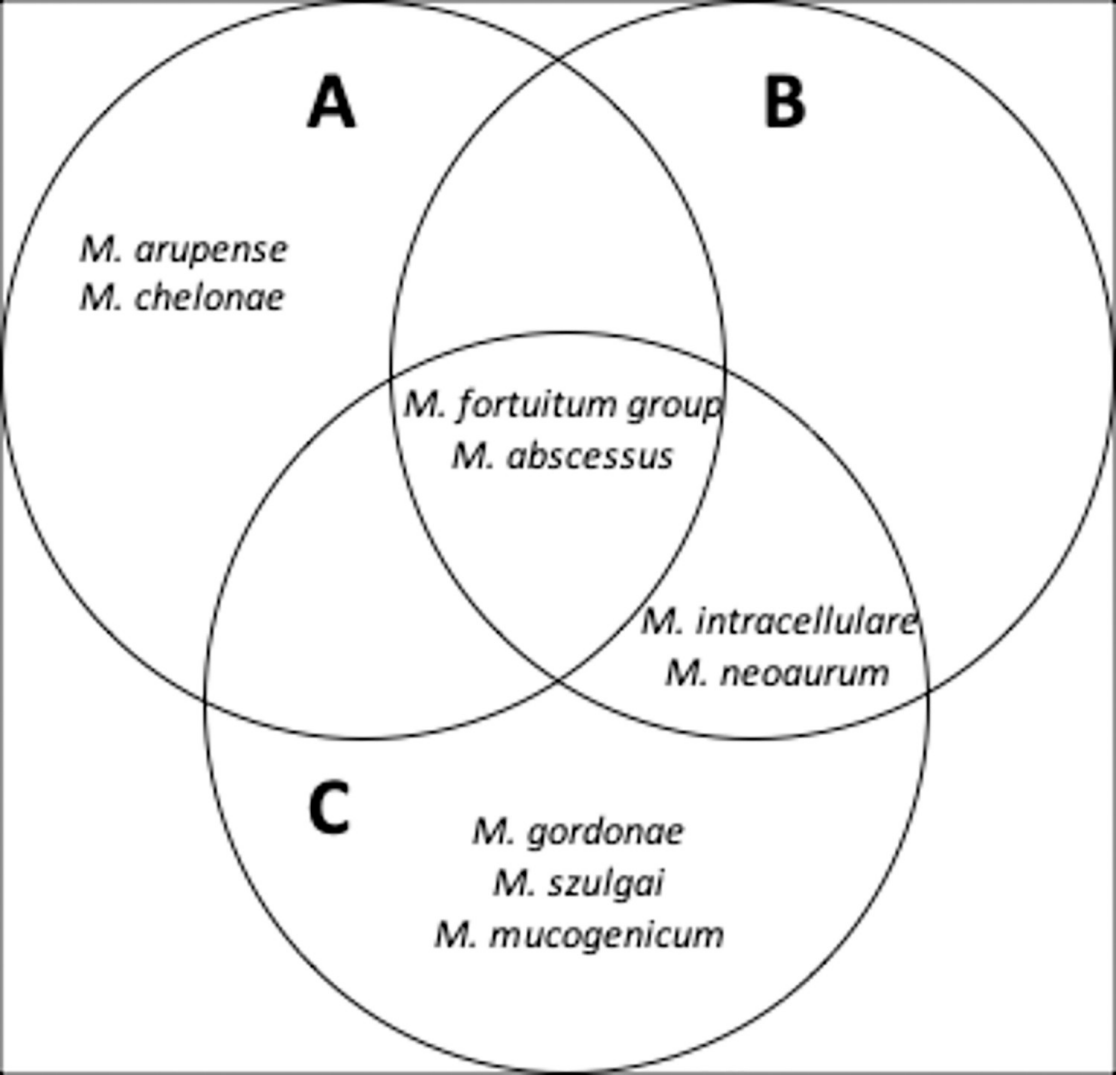
Distribution of NTM species in Java. (A) Types of NTM species in Surabaya-East Java. (B) Types of NTM species in Semarang-Central Java. (C) Types of NTM species in Bogor-West Java.

There were 94 NTM isolates, of which 39 were from West Java, 22 from Central Java, and 33 were from East Java. Table 1 shows patient characteristics. After identifying the species of all the isolates, we found nine groups of NTM: *M*. *fortuitum* group 51% (48/94), *M*. *abscessus* 38.3% (36/94), *M*. *intracellulare* 3.1% (3/94), *M*. *neoaurum* 2.1% (2/94), *M*. *chelonae* 1.1% (1/94), *M*. *gordonae* 1.1% (1/94), *M*. *szulgai* 1.1% (1/94), *M*. *mucogenicum* 1.1% (1/94), and *M*. *arupense* 1.1% (1/94). The *M*. *fortuitum* group comprises all the strains of *M*. *fortuitum* including *M*. *alvei*, *M*. *farcinogenes*, *M*. *fortuitum*, *M*. *fortuitum ssp fortuitum*, *M*. *houstonense*, *M*. *peregrinum*, *M*. *porcinum*, and *M*. *senegalense*. Vitek MS identified all of these species in the *M*. *fortuitum* group [19]. The data revealed differences in the distribution of NTM species in West, Central, and East Java. The *M*. *fortuitum* group and *M*. *abscessus* were found in West Java, Central Java, and East Java. The *M*. *fortuitum* group was predominant in West and Central Java, whereas *M*. *abscessus* was predominant in East Java. Isolates originating from West Java showed the greatest variation in NTM species, with seven species. The distribution of the NTM species is shown in Fig 1.

**Table 1:** Patients’ characteristics.

Characteristics	Category	Bogor (West Java)		Semarang (Central Java)		Surabaya (East Java)		Total	
		n	%	n	%	n	%	n	%
Gender	Male	15	38.5	14	63.6	19	57.6	48	51.1
	Female	24	61.5	8	36.4	14	42.4	46	48.9
Age	15–24 yo	2	5.1	3	13.6	7	21.2	12	12.8
	25–64 yo	36	92.3	19	86.4	24	72.7	79	84.0
	≥65 yo	1	2.6	0	0	2	6.1	3	3.2

### Susceptibility assay

Amikacin was the most effective antibiotic against the *M*. *fortuitum* group and was active against 91.7% of all *M*. *fortuitum* group isolates, followed by moxifloxacin (83.3%), ciprofloxacin (70.8%), trimethoprim/sulfamethoxazole (35.4%), doxycycline (22.9%), clarithromycin (18.8%), and imipenem (2%). Amikacin was the most effective antibiotic against 83.3% of all *M*. *abscessus* isolates, followed by clarithromycin (41.7%), moxifloxacin (13.9%), trimethoprim/sulfamethoxazole (11.1%), ciprofloxacin (5.6%), imipenem (2.8%), and doxycycline (0%) (Tables 2 and 3).

**Table 2:** Test results of *M*. *fortuitum* group susceptibility assay to various antibiotics.

Antibiotics	Isolate origin	Susceptible		Non-susceptible	
		n	%	n	%
Ciprofloxacin	Bogor (West Java)	13	68	6	32
	Semarang (Central Java)	11	79	3	21
	Surabaya (East Java)	10	67	5	33
	Total	34	70.8	14	29.2
Moxifloxacin	Bogor (West Java)	17	89	2	11
	Semarang (Central Java)	12	86	2	14
	Surabaya (East Java)	11	74	4	26
	Total	40	83.3	8	16.7
Clarithromycin	Bogor (West Java)	2	10	17	90
	Semarang (Central Java)	1	7	13	93
	Surabaya (East Java)	6	40	9	60
	Total	9	18.8	39	81.2
Amikacin	Bogor (West Java)	18	95	1	5
	Semarang (Central Java)	14	100	0	0
	Surabaya (East Java)	12	80	3	20
	Total	44	91.7	4	8.3
Imipenem	Bogor (West Java)	0	0	19	100
	Semarang (Central Java)	0	0	14	100
	Surabaya (East Java)	1	7	14	93
	Total	1	2	47	98
Trimethoprim/ sulfamethoxazole	Bogor (West Java)	5	26	14	74
	Semarang (Central Java)	5	36	9	64
	Surabaya (East Java)	7	47	8	53
	Total	17	35.4	31	64.6
Doxcycyclin	Bogor (West Java)	4	21	15	79
	Semarang (Central Java)	2	14	12	86
	Surabaya (East Java)	5	33	10	67
	Total	11	22.9	37	77.1

**Table 3:** Test results of *M*. *abscessus* susceptibility assay to various antibiotics.

Antibiotics	Isolate origin	Susceptible		Non-susceptible	
		n	%	n	%
Ciprofloxacin	Bogor (West Java)	1	6	14	94
	Semarang (Central Java)	1	20	4	80
	Surabaya (East Java)	0	0	16	100
	Total	2	5.6	34	94.4
Moxifloxacin	Bogor (West Java)	2	13	13	87
	Semarang (Central Java)	1	20	4	80
	Surabaya (East Java)	2	12	14	88
	Total	5	13.9	31	86.1
Clarithromycin	Bogor (West Java)	4	27	11	73
	Semarang (Central Java)	2	40	3	60
	Surabaya (East Java)	9	56	7	44
	Total	15	41.7	21	58.3
Amikacin	Bogor (West Java)	14	93	1	7
	Semarang (Central Java)	5	100	0	0
	Surabaya (East Java)	11	69	5	31
	Total	30	83.3	6	16.7
Imipenem	Bogor (West Java)	1	6	14	94
	Semarang (Central Java)	0	0	5	100
	Surabaya (East Java)	0	0	16	100
	Total	1	2.8	35	97.2
Trimethoprim/ sulfamethoxazole	Bogor (West Java)	2	13	13	87
	Semarang (Central Java)	0	0	5	100
	Surabaya (East Java)	2	13	14	87
	Total	4	11.1	32	88.9
Doxcycyclin	Bogor (West Java)	0	0	15	100
	Semarang (Central Java)	0	0	5	100
	Surabaya (East Java)	0	0	16	100
	Total	0	0	36	100

Among the isolates capable of forming biofilms, *M*. *fortuitum* group showed good susceptibility to amikacin (95.2%) and moxifloxacin (85.7%). Among the isolates unable to form biofilms, the *M*. *fortuitum* group showed good susceptibility to amikacin (88.9%) and moxifloxacin (81.5%). Meanwhile, for *M*. *abscessus*, all isolates capable of forming biofilms and unable to form biofilms showed good susceptibility to amikacin at 90.9% and 80%, respectively (Tables 4 and 5).

**Table 4:** Antibiotic susceptibility and biofilm formation in the *M*. *fortuitum* group.

Antibiotics	Biofilm producer (n = 21)				Non-biofilm producer (n = 27)			
	Susceptible		Non-susceptible		Susceptible		Non-susceptible	
	n	%	n	%	n	%	n	%
Ciprofloxacin	14	66.7	7	33.3	20	74.1	7	25.9
Moxifloxacin	18	85.7	3	14.3	22	81.5	5	18.5
Clarithromycin	2	9.5	19	90.5	7	25.9	20	74.1
Amikacin	20	95.2	1	4.8	24	88.9	3	11.1
Imipenem	0	0	21	100	1	3.7	26	96.3
Trimethoprim/ sulfamethoxazole	6	28.6	15	71.4	11	40.7	16	59.3
Doxycycline	2	9.5	19	90.5	9	33.3	18	66.7

**Table 5:** Antibiotic susceptibility and biofilm formation in *M*. *abscessus*.

Antibiotics	Biofilm producer (n = 11)				Non-biofilm producer (n = 25)			
	Susceptible		Non-Susceptible		Susceptible		Non-susceptible	
	n	%	n	%	n	%	n	%
Ciprofloxacin	2	18.2	9	81.8	0	0	25	100
Moxifloxacin	2	18.2	9	81.8	3	12.0	22	88.0
Clarithromycin	5	45.5	6	54.5	10	40	15	60.0
Amikacin	10	90.9	1	9.1	20	80	5	20.0
Imipenem	0	0.0	11	100	0	0.0	25	100
Trimethoprim/ sulfamethoxazole	2	18.2	9	81.8	2	8.0	23	92.0
Doxycycline	0	0	11	100	0	0.0	25	100

### Biofilm assay

**Fig 2:**
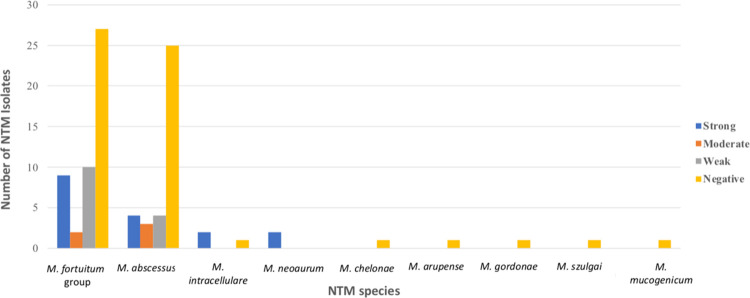
Ability to form biofilms by various NTM species. Most of the tested isolates of the *M*. *fortuitum* group and *M*. *abscessus* were unable to form biofilms. The number of isolates in the *M*. *fortuitum* group that formed a strong biofilm was higher than that of *M*. *abscessus*. Most other species could not form biofilms.

**Fig 3:**
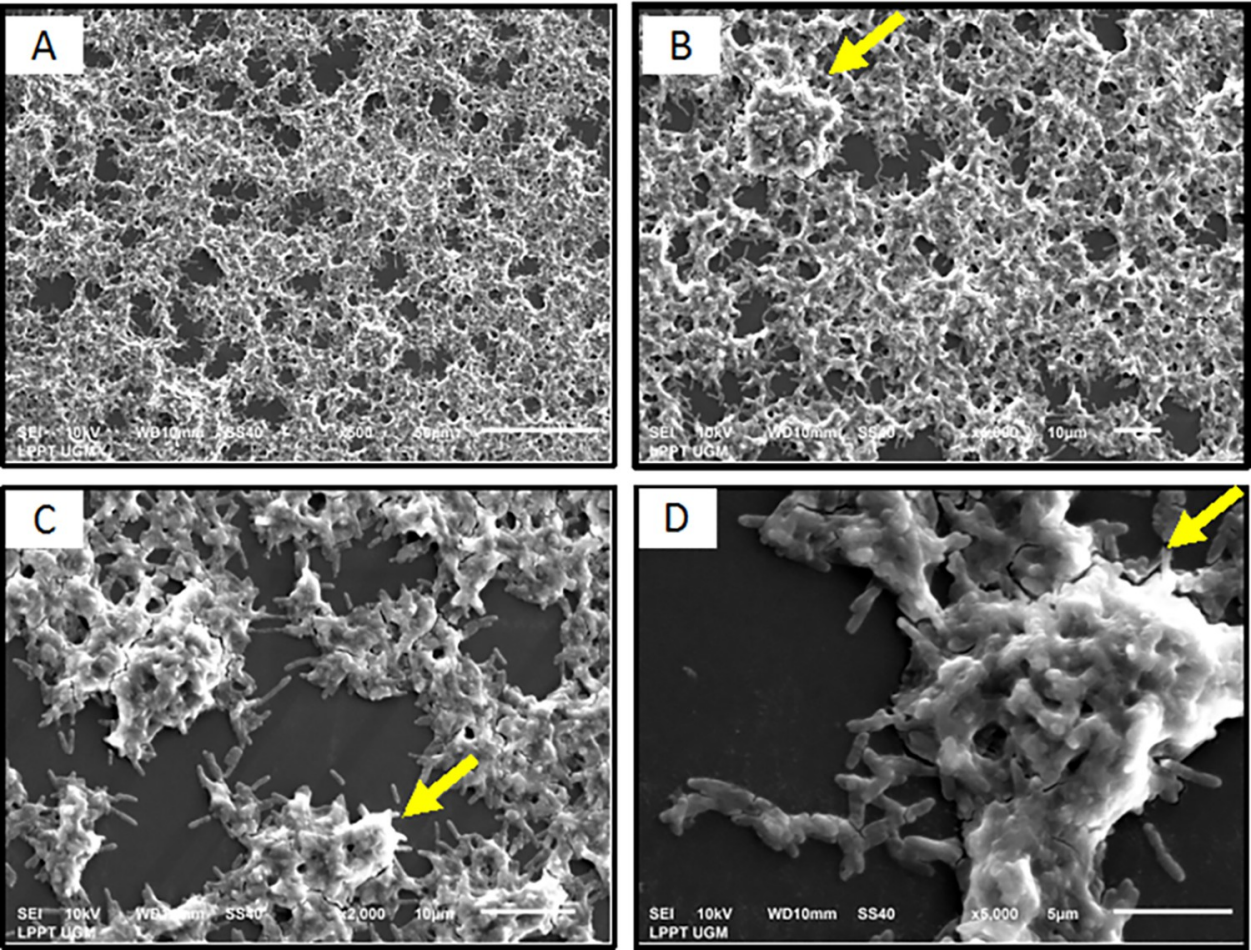
The structure of biofilm cells in the *M*. *fortuitum* group on observation by SEM on the 7^th^ day of incubation. Yellow arrows indicate extra-cellular polymer substance (EPS) formation. (A) Observation at 500× magnification. (B) Observation at 1000× magnification. (C) Observation at 2000× magnification. (D) Observation at 5000× magnification.

**Fig 4:**
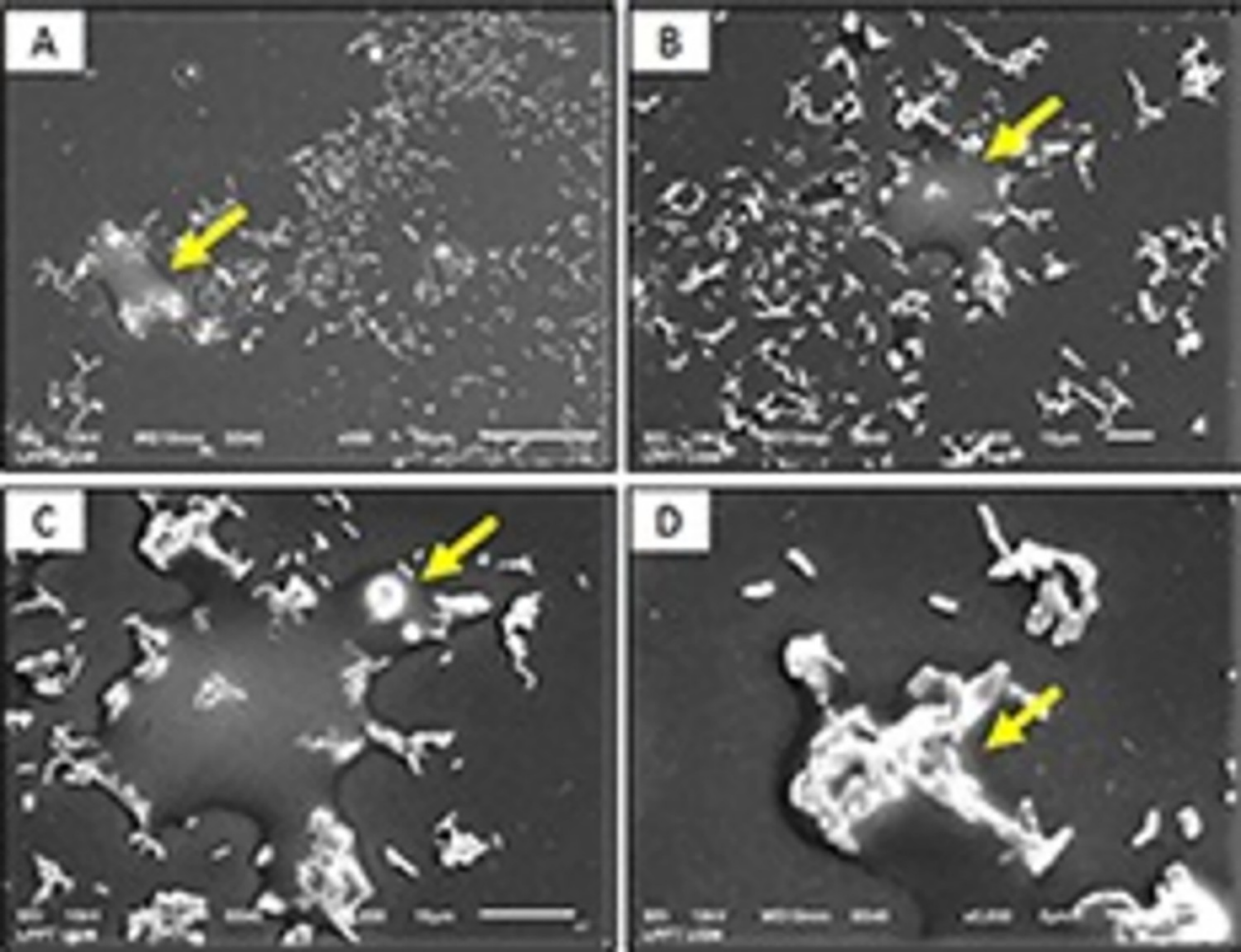
The cell structure of the biofilm in *M*. *abscessus* on observation by SEM on the 7^th^ day of incubation. Yellow arrows indicate extra-cellular polymer substance (EPS) formation. (A) Observation at 500× magnification. (B) Observation at 1000× magnification. (C) Observation at 2000× magnification. (D) Observation at 5000× magnification.

Among the 48 isolates of the *M*. *fortuitum* group, 21 (43.8%) could form biofilms, and 27 (56.2%) were unable to form biofilms. Among the 36 isolates of *M*. *abscessus*, 11 (30.6%) could form biofilms, and 25 (69.4%) were unable to form biofilms. The number of isolates capable of forming a strong biofilm was 18.75% (9/48) in the *M*. *fortuitum* group., while it was only 11.11% (4/36) for *M*. *abscessus*, (Fig 2). Based on the chi-square test results, we found a significant difference in the ability to form biofilms in the *M*. *fortuitum* group and *M*. *abscessus* (*p* < 0.001). The number of isolates in the *M*. *fortuitum* group that formed a strong biofilm was higher than that of *M*. *abscessus*. The results of SEM observations on the 7^th^ day of incubation showed that the *M*. *fortutum* group isolates formed biofilms better than *M*. *abscessus* (Figs 3 and 4).

### Sliding motility assay

**Fig 5:**
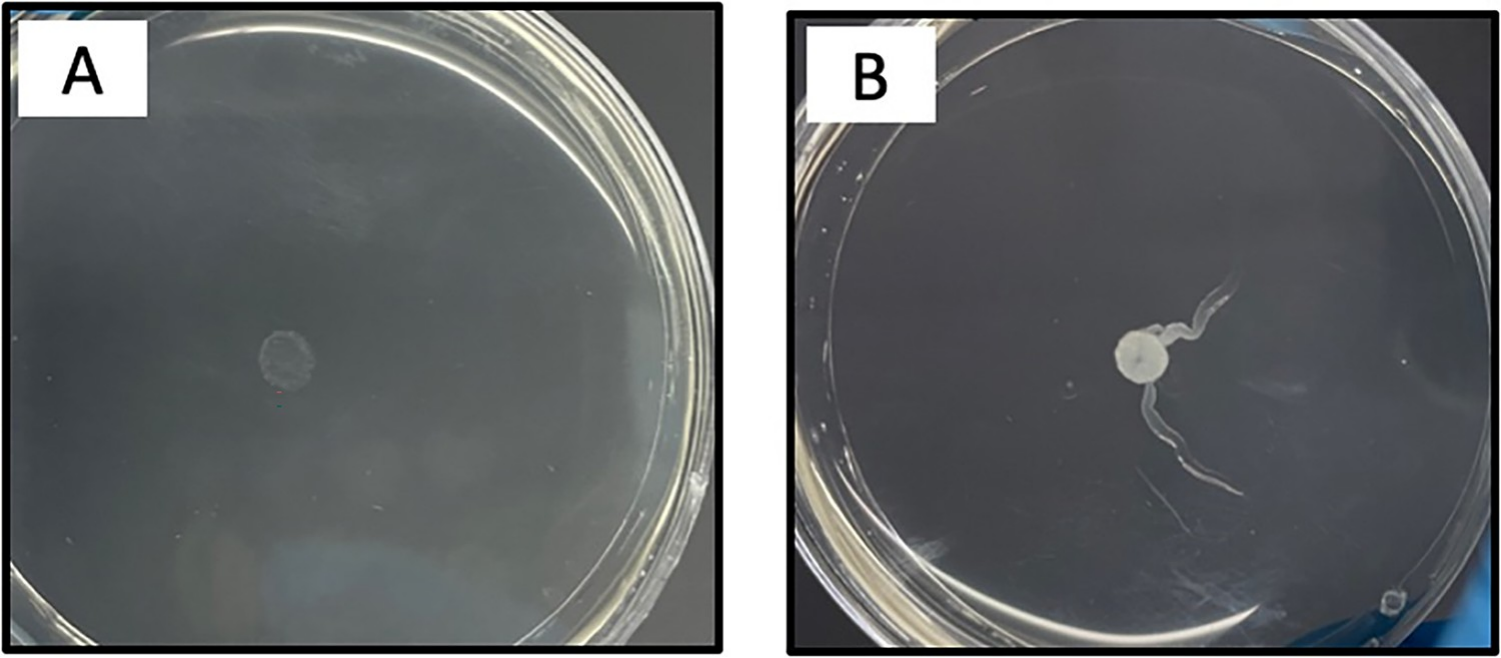
Sliding motility of *M*. *abscessus*. (A) Observations on the 8^th^ day of incubation. (B) Observations on the 16^th^ day of incubation.

Of the 31 isolates (16 isolates of the *M*. *fortuitum* group and 15 isolates of *M*. *abscessus*), 19 (61.3%) could perform sliding motility, and 12 (38.7%) were unable perform sliding motility. Among the isolates capable of sliding motility, 42.1% (8/19) could form biofilms, and 57.9% (11/19) were unable to form biofilms. The *M*. *fortuitum* group and *M*. *abscessus* showed the longest displacements of 24.75 mm and 17.45 mm, respectively, observed on day 16 (Table 6). The results of the analysis using the Kruskal–Wallis test showed that there was no significant difference in sliding motility between days 4, 8, 12, and 16 in both the *M*. *fortuitum* group (*p* = 0.71) and *M*. *abscessus* (*p* = 0.17). The analysis results using the Mann–Whitney test showed that there was no significant difference in the ability to perform sliding motility between the *M*. *fortuitum* group and *M*. *abscessus* (*p* = 0.57). Fig 5 shows the sliding motility of *M*. *abscessus* on days 8 and 12.

**Table 6:** Test results of sliding motility.

NTM Species Name (n)	Diameter of sliding motility in mm (mean ± SD)			
	Day-4	Day-8	Day-12	Day-16
*M*. *fortuitum group* (16)	4.66 **±** 5.57	6.04 **±** 6.02	7.04 **±** 7.36	7.92 **±** 8.17
*M*. *abscessus* (15)	3.05 **±** 2.65	4.98 **±** 4.40	6.11 **±**5.62	6.88 **±** 6.50

NTM: nontuberculous mycobacteria, SD: standard deviation

### Adherence and invasion assay

The *M*. *fortuitum* group showed better adhesion and invasion than did *M*. *abscessus*. This was indicated by the number of *M*. *fortuitum* group bacteria capable of adhesion and invasion, which was higher than that of *M*. *abscessus*, at 440 **±** 81.55 and 348.33 **±** 128.65, respectively.

## Discussion

This study revealed differences in the distribution of NTM species between West Java, Central Java, and East Java, Indonesia. The two most common species found in this study were *M*. *fortuitum* group and *M*. *abscessus*. The susceptibility of *M*. *abscessus* was lower than that of *M*. *fortuitum* group. Other antibiotics in the *M*. *fortuitum* group that had a susceptibility greater than 50% were moxifloxacin (83.3%) and ciprofloxacin (70.8%), while there were no other antibiotics with a susceptibility above 50% in *M*. *abscessus*, with the exception of amikacin. Amikacin was the most effective antibiotic against *M*. *fortuitum* group and *M*. *abscessus*. The data from this study showed that both the *M*. *fortuitum* group and *M*. *abscessus* were capable of forming biofilms. The isolates of the *M*. *fortuitum* group formed biofilms better than those of the *M*. *abscessus*. The results showed that the number of bacteria capable of adhesion and invasion was higher in the *M*. *fortuitum* group than in the *M*. *abscessus*. There was no significant difference between the *M*. *fortuitum* group and *M*. *abscessus* in terms of the ability for sliding motility.

These results are similar to those of other publications showing significant differences in the distribution of NTM clinical isolate species in various countries and geographic areas [27]. These differences in species across regions affect the incidence of infection and clinical manifestations of NTM-PD. This is because each type of NTM species has a different ability to cause lung infections in humans [28]. Differences in environmental factors are very important in terms of the difference in the distribution of NTM in various regions and their relationship with clinical manifestations and therapeutic outcomes [29]. The average local annual temperature affects the composition of RGM found in an area [30,31]. Temperature conditions in the three provinces studied were quite varied. On the island of Java, the regions, arranged in increasing order of average annual temperatures, are West Java, Central Java, and East Java [32]. The data from this study showed that the *M*. *fortuitum* group is the major NTM species in West Java and Central Java, while *M*. *abscessus* is an NTM species commonly found in Surabaya, East Java. *M*. *abscessus* was most commonly found in areas with high mean temperature. This is in contrast to the results of a study conducted in Japan, where *M*. *abscessus* was found in areas with lower temperatures. At lower temperatures, *M*. *abscessus* showed higher activity than *M*. *fortuitum* and *M*. *chelonae* [30]. Hoefsloot et al. (2013) showed that *M*. *fortuitum* and *M*. *abscessus* are the two most common RGM-type NTM species found in clinical isolates worldwide and are the most common cause of infection. Other species of RGM are found only sporadically [33]. Han et al. (2008) also showed that approximately 90% of RGM infections are caused by *M*. *fortuitum* complex, *M*. *abscessus*, and *M*. *mucogenicum* [34].

*M*. *abscessus* complex resistant to various antibiotics and is only sensitive *in vitro* to parenteral agents, such as amikacin, cefoxitin, imipenem, and sometimes to oral macrolides [11]. Similar results were reported in a study by Cho et al. (2019), who found that *M*. *abscessus* is highly sensitive to amikacin [35]. Amikacin, cefoxitin, and clarithromycin have been established as standard antibiotics for infections caused by *M*. *abscessus* [36]. The study of antibiotic susceptibility of the NTM genus by Nuryastuti et al. (2018) showed somewhat different results. Clarithromycin was the most effective antibiotic against NTM isolates followed by gentamicin, kanamycin, levofloxacin, ofloxacin, and ceftriaxone. The isolates studied were resistant to cotrimoxazole and amoxicillin [37]. Although amikacin still shows good efficacy against *M*. *abscessus*, the study conducted by Park et al. (2020) showed that there is already a fairly high resistance to aminoglycoside antibiotic groups; in this case, to amikacin [38]. Resistance to aminoglycosides is related to mutations in the *rrs* gene [39]. Antimicrobial resistance can be classified as acquired or intrinsic [35]. Acquired resistance is caused by gene mutations [40] and is related to the long-term use of antibiotics during NTM therapy [41]. Meanwhile, intrinsic resistance can be caused by, among other factors, their thick impermeable hydrophobic cell wall, which acts as a barrier, and the formation of biofilms [41].

The results of this study showed that the number of antibiotic-resistant isolates of the *M*. *fortuitum* group and *M*. *abscessus* in the biofilm producer group was higher than that in the non-biofilm producer group. Greendyke et al. (2008) studied the susceptibility of *M*. *abscessus* to the antibiotics amikacin, clarithromycin, and cefoxitin. The results showed that in the planktonic state, all antibiotics had MIC values that indicated sensitive results, but the minimum bactericidal concentration (MBC) of amikacin and clarithromycin showed higher values than those of MIC. In contrast to amikacin and clarithromycin, cefoxitin has no bactericidal effect. When *M*. *abscessus* was grown as biofilm, amikacin showed moderate effectiveness against *M*. *abscessus*, but clarithromycin was only minimally effective. Like as in the planktonic state, cefoxitin had only bacteriostatic effect against *M*. *abscessus*. This study showed that *M*. *abscessus* with mature biofilms was in the stationary-phase and clarithromycin was inactivated in this condition [36]. Another study on control strains of *M*. *chelonae* ATCC 35752 and *M*. *fortuitum* ATCC 49404 conducted by Aung et al. (2015) also showed that the biofilm on mycobacteria is quite resistant to conventional antibiotics commonly used. A new therapeutic strategy in the form of a combination of DNase with antibiotics is more effective than using antibiotics alone [42].

Therapy against biofilms is more effective when antibiotics are administered at an early stage of biofilm formation [17]. This study showed that isolates from the *M*. *fortuitum* group formed biofilms better than *M*. *abscessus*. The research results follow those of Sousa et al. (2015), who showed that *M*. *smegmatis* and *M*. *fortuitum* were the two best biofilm-forming species [18]. Hydrophobic mycobacterial surfaces [43] are thought to play a role in biofilm formation [44]. The ability to form biofilms is not consistent among clinical RGM isolates. However, it appears to be related to the capacity of the strain to cause infection [45].

Infection begins with adhesion to host tissues and cells. The hydrophobicity of the cell wall is a factor that affects the ability to adhere [46]. The results of this study showed that the number of bacteria capable of adhesion and invasion was higher in the *M*. *fortuitum* group than in *M*. *abscessus*. A previous study showed that the cell surface of *M*. *abscessus* is less hydrophobic than that of *M*. *avium* and *M*. *intracellulare*; therefore, the number of *M*. *abscessus* cells capable of adhesion can be expected to be lower than that of *M*. *avium* and *M*. *intracellulare* [44]. Other studies have shown that the ability to form biofilms also affects bacteria’s ability to invade epithelial cells [47]. This research supports the results of the present study, in which it is known that the *M*. *fortuitum* group can form biofilms more quickly and has better adhesion and invasion abilities than do *M*. *abscessus*.

Recht et al. (2001) have shown that glycopeptidolipids, are not only important in the attachment stage during biofilm formation, but also play an important role in the ability to perform sliding motility in *M*. *smegmatis* [48]. Previous studies have also reported an association between biofilm formation and the sliding motility of *Mycobacterium spp* [49]. However, there are several concerns about this relationship [45]. Studies by Martin-de-Hijas et al. (2009) showed that 61.8% of isolates with positive biofilms and 55.6% of isolates with negative biofilms exhibited sliding motility [45].

The results of this study describe the distribution of NTM species in Java. With these data, although clinicians did not identify NTM at the species level, they were able to determine the most appropriate empirical antibiotic based on the results of this study. Scientific information regarding biofilm formation in NTM species can assist clinicians in considering the administration of antibiofilm therapy regimens. The results of this study are expected to provide new management strategies for increasing the healing rate of patients with NTM-PD infection on Java. Based on the results of this study, clinicians should be more aware of the significance of NTM infections. In addition, they can increase awareness and concern for identifying NTM at the species level and testing its susceptibility to various antibiotics.

The strength of this study is that it was a multicenter study conducted in Java. Samples were obtained from clinical isolates from three pulmonary TB referral centers in West Java, Central Java, and East Java, Indonesia. This study also identified NTM at the species level and is the first study conducted in Indonesia to do so. In addition, we analyzed various virulence factors associated with NTM species. The identification of NTM species was performed using MALDI-TOF MS, which is the first time that this has been carried out in Indonesia. A limitation of this study was that the susceptibility test was performed on planktonic bacteria; therefore, the relationship between biofilm capability and antibiotic susceptibility could not be directly described. Further studies are needed to determine the susceptibility of NTM to other types of antibiotics. In addition, it is necessary to conduct studies that analyze the relationship between NTM and the clinical manifestations in patients. Unfortunately, we do not know whether every individual from whom a sample was sent to each TB referral center meets the criteria for NTM-PD.

In conclusion, our findings showed that *M*. *fortuitum* group and *M*. *abscessus* are the most common NTM found in Java, Indonesia. A significant difference was observed in the ability of the two species to form biofilms. Although the *M*. *fortuitum* group was able to form a biofilm better than *M*. *abscessus*, antibiotic susceptibility tests showed that the *M*. *fortuitum* group was less resistant than *M*. *abscessus*. In contrast, the *M*. *fortuitum* group showed better adhesion and invasion abilities than those of *M*. *abscessus*. The data showed no significant differences in the ability to perform sliding motility. Thus, the ability to form biofilms is directly proportional to their ability to the adhere and invade.

## Supporting information

S1 Data Excel spreadsheet containing the underlying numerical data for Figs 1 and 2, and also Tables 1,2,3,4,5 and 6. (XLSX)

S1 Text Laboratory protocols for investigating NTM characteristics. http://dx.doi.org/10.17504/protocols.io.ewov1o84ylr2/v1.
(DOCX)
