# Supplementary material for: Characteristics of clinical isolates of nontuberculous mycobacteria in Java-Indonesia: A multicenter study
Source: PLoS Negl Trop Dis. 2022 Dec 27;16(12):e0011007. doi: 10.1371/journal.pntd.0011007 (PMC9829163; doi:10.1371/journal.pntd.0011007)
Supplement: S1 Text — http://dx.doi.org/10.17504/protocols.io.ewov1o84ylr2/v1. (DOCX) [file pntd.0011007.s002.docx]

LABORATORY PROTOCOLS FOR INVESTIGATING

NTM CHARACTERISTICS

A. NTM species identification

Species identification was performed by using matrix-assisted laser desorption-ionization time-of-flight mass spectrometry (MALDI-TOF MS) (Vitek MS *Mycobacterium*/*Nocardia* Kit, bioMérieux SA, Marcy L'Étoile, France) [1], as follows:

1. NTM colonies were taken from LJ media using a 1 μL loop and put into a 1.5 mL microtube containing 0.5 mm of glass beads and 500 μL of 70% ethanol.
2. Vortex was carried out for 15 minutes and then incubated vertically at room temperature for 10 minutes.
3. Vortex was carried out again, transferred into 2 ml of round-bottomed microtube, and then centrifuged at a speed of 10.000-14.000 g for 2 minutes.
4. Supernatant was removed, 10 μL of 70% formic acid was added to the pellet, and then vortex was carried out until homogeneous.
5. 10 μL of acetonitrile was added, and then vortex was carried out until homogeneous.
6. Centrifugation was carried out at a speed of 10.000-14.000 g for 2 minutes.
7. 1 μL of supernatant was taken, placed on the target spot on the slide, and allowed to dry completely.
8. 1 μL of CHCA matrix (bioMérieux SA, Marcy L'Étoile, France) was added and allowed to dry until the matrix and isolate crystallized.
9. Slide was loaded into the Vitek MS machine (bioMérieux SA, Marcy L'Étoile, France) and the sample number was added.
10. The results were read using a computer connected to Vitek MS machine (bioMérieux SA, Marcy L'Étoile, France). The identification test results will appear on the monitor in the form of the NTM species name.

B. Susceptibility assay

The test procedure was performed following the method of Li et al. (2013), with slight modifications [2]:

1. Determination of the concentration of the stock of antibiotics was conducted through the dilution process. The dilution was carried out based on the content of each antibiotic used to obtain a final concentration of 1000 μg/mL.
2. 0.1 mL of Middlebrook 7H9 supplement (7H9-S) (7H9 broth +10% ADC + 0.5% glycerol) was added to each well microdilution plate using a pipette, and one column was left for negative control with a volume of 0.2 mL.
3. 0.1 mL of antibiotic was added to each column, and serial dilution was carried out in each subsequent column, leaving 1 column for positive control.
4. Bacterial colonies were taken from 3-5 colonies of LJ medium and transferred to the saline solution using a tube until it reached turbidity of 0.5 McFarland (1-2 × 10^8^ CFU/mL).
5. Diluted suspension of 0.5 McFarland (1-2 × 10^8^ CFU/mL) was carried out using Middlebrook 7H9 supplement (7H9-S) in a ratio of 1:20 so that the concentration became 5-10 x 10^6^ CFU/mL, then 0.1 mL was added into each appropriate column, except for the sterile control column.
6. In each well, around the microdilution plate, saline was added to keep the atmosphere moist.
7. Microdilution plate was wrapped with plastic or plastic tape to prevent dryness.
8. Incubation was done at a temperature of 35 ± 2°C for 7 days.
9. Observation of growth was conducted through the growth of bacteria in wells containing antibiotics compared to growth controls.
10. The test was repeated 3 times, and the MIC value was determined for each test. The MIC breakpoints of the drugs were interpreted according to the Clinical and Laboratory Standards Institute guidelines (M24-A2) [3].
11. Determination was carried out into susceptible (S), intermediate (I), or resistant (R) groups based on the MIC criteria according to Table 1.

Table 1. MIC values for each type of antibiotic

| Antibiotics | MIC Category (μg/mL) | | | |
| --- | --- | --- | --- | --- |
|  | Susceptible | Intermediate | | Resistant |
| Ciprofloxacin (CIP) | ≤1 | | 2 | ≥4 |
| Moxifloxacin (MFX) | ≤1 | | 2 | ≥4 |
| Clarithromycin (CLR) | ≤2 | | 4 | ≥8 |
| Amikacin (AMK) | ≤16 | | 32 | ≥64 |
| Imipenem (IPM) | ≤4 | | 8-16 | ≥32 |
| Trimethoprim/sulfamethoxazole (SOX) | ≤2/38 | | - | ≥4/76 |
| Doxycyclin (DOX) | ≤1 | | 2-4 | ≥8 |

Note: For SOX antibiotic, MIC values between 2/38 and 4/76 are included in category intermediate.

C. Biofilm assay:

The quantitative biofilm testing procedure was performed according to the method of Hassan et al. (2011) with slight modifications [4], as follows:

1. NTM isolates were grown in Middlebrook 7H9 medium for 24 hours at 37°C.
2. Each well of a 96-well microtiter polystyrene plate was filled with 198 μL of Middlebrook 7H9 medium (except the wells in column 1 and 12, row A and H).
3. 2 μL of NTM isolate suspension was added into each column filled with media and left 1column for negative control. In the negative control column, isolate *S. epidermidis* ATCC 12228 was added.
4. The wells in column 1 and 12, row A and H, were filled with saline to keep the atmosphere moist. The biofilm formation test using 96-well microtiter polystyrene plate is shown in Fig 1.
5. Incubation was completed at 37°C for 7 days.
6. The wells were washed three times with 200 μL PBS.
7. Adherent cells were stained with 0.1% crystal violet (50 μL), and then washed with distilled water 3 times (using a pipette).
8. The paint was resuspended with 200 μL of 5% isopropanol acid, and then the absorbance was observed at λ 595 nm. The results were read using a microplate reader (Bio-Rad Benchmark, New Delhi).
9. The bacterial OD value was calculated based on the absorbance value for 6 repetitions carried out in the B-G well. The OD value of bacteria was obtained by calculating the average absorbance value of at least 3 almost the same.
10. ODc value was calculated, where ODc = Average OD of the negative control + 3× SD of negative control.
11. Biofilm production was evaluated according to the criteria described by Stepanovic et al. (2007) [5]. The results were interpreted into categories of strong, moderate, weak, and negative, as shown in Table 2.

Table 2. Interpretation of the results of biofilm formation

| No | Bacterial OD value and ODc value | Interpretation |
| --- | --- | --- |
| 1. | Bacterial OD > 4× Odc value | Strong |
| 2. | 2× Odc value < Bacterial OD < 4× Odc value | Moderate |
| 3. | ODc < Bacterial OD < 2× Odc value | Weak |
| 4. | Bacterial OD ≤ Odc value | Negative |


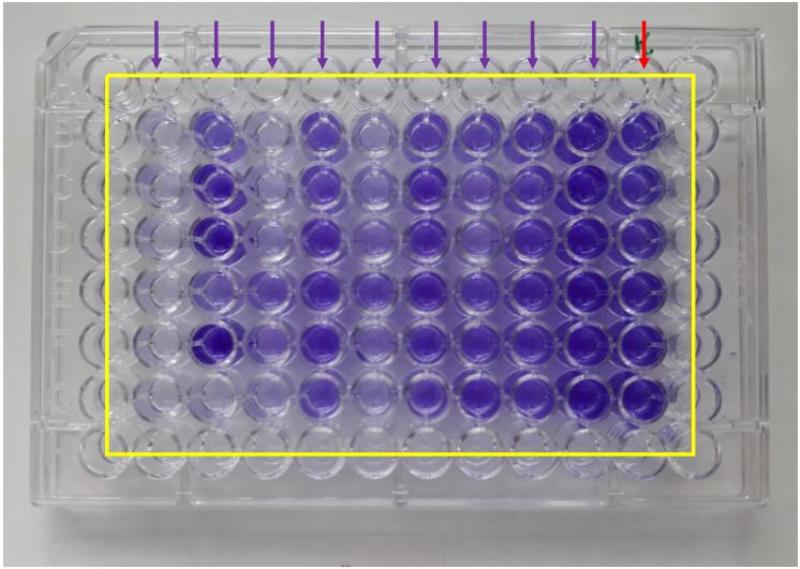


Figure 1. Biofilm testing on a 96-well microplate. The picture above shows the biofilm mass measurement test results on a 96-well microplate. The yellow line indicates the well filled with saline. Red arrows indicate columns for negative controls, and purple arrows indicate columns for samples. The negative control bacteria used was *S. epidermidis* ATCC 12228. The results were read using a microplate reader (Bio-Rad Benchmark, New Delhi).

D. Observation of NTM biofilm cell structure using scanning electron microscopy (SEM).

Scanning electron microscopy (SEM) observations were performed by following the method described by Nuryastuti et al. (2018) with slight modifications [6], with following steps:

1. Bacterial suspension was prepared equivalent to 1 Mc Farland.
2. 20 mL bacterial suspension was put into a 12-well microtiter plates (Corning Costar, Sigma-Aldrich, Missouri, USA) filled with polyvinyl chloride coverslips (0.13–17 mm thick and 13 mm in diameter) and 198 μL of Middlebrook 7H9 media.
3. Incubation was carried out at 35-37°C for 7 days.
4. Wells overgrown with biofilm were wash with PBS (0.1 M and pH 7.2) 2 times.
5. The coverslips were taken and rehydrated using ethanol serially (70% for 10 minutes and then 96% for 10 minutes).
6. Ethanol was discarded, allowed to dry overnight at room temperature.
7. The coverslips were taken and coated twice with platinum vanadium using an ion sputter (BAL-Tec SCD 005) for 11 seconds, after which the coverslips were glued to double-side carbon tape to be observed using SEM (JEOL JED-2300, Japan).

E. Sliding motility assay:

The sliding motility test was performed as described by Esteban et al. (2008) [7]:

1. Sliding motility media was prepared, consisting of Middlebrook 7H9 medium with 0.3% agar without supplements.
2. Inoculation of NTM was carried out as much as 3 μL at optical density (OD) 600.6 (2.7 × 105 CFU) in the middle of the sliding motility media, and the plate was covered with parafilm.
3. Incubation was carried out at 37°C in a 5% CO_2_ atmosphere for up to 16 days in a humid atmosphere.
4. Measurement of the length of the NTM growth area was carried out on days 4, 8, 12, and 16 using a digital caliper in mm.
5. The sliding motility test was carried out 3 times.
6. The average length of growth was calculated from 3 test repetitions.

F. Ability to perform adhesion and invasion:

The test procedure following the protocol in previous publication by Eijkelkamp et al. (2011) with modifications [8], as follows:

1. A549 cell line (human type II pneumocyte) culture was carried out.
2. Supplement at Dulbecco's modified Eagle’s medium (DMEM; Invitrogen, Australia) was added with 10% fetal bovine serum (FBS; Bovogen, Australia), 100 μg/mL of streptomycin, and 2 mM of L-glutamine.
3. Observation of monolayer cell growth was carried out until it reached >95% confluency.
4. The monolayer cells were washed with PBS, and about 1x10^7^ CFU of NTM bacteria were added to each well.
5. Incubation of A549 cell line (human type II pneumocyte), which had been infected at 37°C for 4 hours, was carried out.
6. The culture medium was removed, and 0.25% trypsin was added to the PBS to remove the A549 cell line (human type II pneumocyte) from the well surface.
7. The A549 cell line (human type II pneumocyte), which had been washed using 200 μL of 0.025% sterile Triton X-100, was lysed.
8. Dilution was carried out for 1000×.
9. 20 μL suspension was put into Middlebrook 7H9 medium to determine the amount of bacterial CFU attached to each well.
10. Incubation was carried out for 10 days.
11. Bacterial colonies were counted.
12. Tests were repeated on each species three times at different times. Each time the test was repeated twice.

**References:**

[1] Luo LL, Cao W, Chen WW, Zhang RR, Jing LJ, Chen HP, et al. Evaluation of the VITEK MS knowledge base version 3.0 for the identification of clinically relevant Mycobacterium species article. Emerg Microbes Infect 2018;7. https://doi.org/10.1038/s41426-018-0120-3.

[2] Li G, Lian LL, Wan L, Zhang J, Zhao X, Jiang Y, et al. Antimicrobial susceptibility of standard strains of nontuberculous mycobacteria by microplate Alamar Blue assay. PLoS One 2013;8:4–9. https://doi.org/10.1371/journal.pone.0084065.

[3] Woods GL, Brown-Elliot BA, Conville PS, Desmond EP, Hall GS, Lin G, et al. Susceptibility Testing of Mycobacteria, Nocardiae, and Other Aerobic Actinomycetes; Approved Standard—Second Edition. CLSI document M24-A2. 31st ed. Wayne, PA: Clinical and Laboratory Standards Institute; 2011.

[4] Hassan A, Usman J, Kaleem F, Omair M, Khalid A, Iqbal M. Evaluation of different detection methods of biofilm formation in the clinical isolates. Braz J Infect Dis 2011;15:305–11.

[5] Stepanovic S, Vukovic D, Hola V, Bonaventura G DI, Djukic S, Ruzicka F, et al. Quantification of biofilm in microtiter plates: overview of testing conditions and practical recommendations for assesment of biofilm production by staphylococci. APMIS 2007;115:891–900. https://doi.org/https://doi.org/10.1111/j.1600-0463.2007.apm_630.x.

[6] Nuryastuti T, Setiawati S, Ngatidjan N, Mustofa M, Jumina J, Fitriastuti D, et al. Antibiofilm activity of (1)-N-2-methoxybenzyl-1,10-phenanthrolinium bromide against Candida albicans. J Mycol Med 2018;28:367–73. https://doi.org/10.1016/j.mycmed.2017.12.010.

[7] Esteban J, Martín-de-hijas NZ, Kinnari TJ, Ayala G, Fernández-roblas R, Gadea I. Biofilm development by potentially pathogenic non-pigmented rapidly growing mycobacteria 2008;8:1–8. https://doi.org/10.1186/1471-2180-8-184.

[8] Eijkelkamp BA, Stroeher UH, Hassan KA, Papadimitrious MS, Paulsen IT, Brown MH. Adherence and motility characteristics of clinical Acinetobacter baumannii isolates. FEM Microbiol Lett 2011;323:44–51. https://doi.org/10.1111/j.1574-6968.2011.02362.x.
